# Treatment Efficacy with a Novel Hyaluronic Acid-Based Hydrogel for Osteoarthritis of the Knee

**DOI:** 10.3390/jpm11040303

**Published:** 2021-04-15

**Authors:** Octav Marius Russu, Tudor Sorin Pop, Andrei Marian Feier, Cristian Trâmbițaș, Zsuzsanna Incze-Bartha, Paul Gabriel Borodi, István Gergely, Sándor-György Zuh

**Affiliations:** 1 Department of Orthopaedics and Traumatology, Clinical County Hospital, 540139 Tîrgu Mureș, Romania; octav@genunchi.ro (O.M.R.); sorintpop@yahoo.com (T.S.P.); drtrambitas@ortopedultau.ro (C.T.); zsuzsanna.incze-bartha@umfst.ro (Z.I.-B.); gergelyistvan@studium.ro (I.G.); zuh.sandor@gmail.com (S.-G.Z.); 2 Faculty of General Medicine, University of Medicine, Pharmacy, Sciences and Technology, 540139 Tîrgu Mureș, Romania; borodi.paul@yahoo.com; 3 Department of Anatomy and Embriology, University of Medicine, Pharmacy, Sciences and Technology, 540139 Tîrgu Mureș, Romania

**Keywords:** hyaluronic acid, osteoarthritis, intra-articular injection

## Abstract

**Background**: Prior trials investigating the treatment of symptomatic osteoarthritis (OA) with hyaluronic-acid-derived products injections have provided optimistic results. The study was directed to assess the effectiveness of an innovative hyaluronic-acid-based hydrogel (Hymovis^®^) in the treatment of symptomatic knee OA. **Methods**: A prospective, single-center, clinical trial was performed. Thirty-five patients with degenerative knee OA were included. Inclusion criteria were: age between 45–80, radiographic Kellgren grade II or III osteoarthritis, minimum 35 mm score on the Visual Analogue Scale (VAS), pain for at least 6 months and agreement to participate in the study. Patients received two injections at a one-week interval. The evaluator assessed the patients using the Western Ontario and McMaster University Osteoarthritis Index (WOMAC) and VAS. Evaluation was performed before, at 2 and 6 months after the injections. **Results**: A significant improvement on the WOMAC Index pain subscale was observed at 6 months after the injection. At two months, pain subscale score decreased from 10.34 to 9.34. At six months, a significant decrement in pain parameters compared to baseline was observed (from 10.34 to 7.72; *p* = 0.0004). Median points on VAS significantly ameliorated after 6 months (from 74.2 to 57.3 cm; *p* < 0.0001). Regarding physical function, a statistically significant difference compared to baseline was observed at the end of the study (from 29.74 to 25.18; *p* = 0.0025). WOMAC Index stiffness component did not differ from baseline at any time during follow-up. **Conclusions**: Pain relief installed with a delayed on-set but had a prolonged duration. The novel hyaluronic acid-based hydrogel (Hymovis^®^) had effective results, particularly after six months post-injections and offers a therapeutic advancement in the treatment of moderate to severe osteoarthritis.

## 1. Introduction

Knee osteoarthritis (OA) is a common chronic joint disorder and a leading cause of disability among the elder population [1]. Pain decrease, improvement in joint mobility and functional impairment reduction are the main objectives in the treatment of knee OA. Although there is a high demand for medication that prevents or reduces the development of cartilage destruction in OA, no such treatment has been discovered so far [2]. Using a wide variation of both oral and injected conservative therapies, such as analgesics, non-steroidal anti-inflammatory drugs (NSAIDs) and intra-articular injections, might prolong the period until surgery for most patients [3,4,5].

Most of the oral medications have weak or no effects when treating pain associated with knee OA. Acetaminophen is proven to have no effects on pain control irrespective of dose [6] and some of the NSAIDs are well known in the literature for their adverse reactions causing toxicity (gastric and cardiovascular) [7,8,9,10] and are being recognized as responsible for most of the iatrogenic pathology [11]

It was also described that usage of oral opioids (e.g., Tramadol) has a small benefit compared to their potential adverse events [12]. As elderly patients have a higher incidence of knee OA, they are also considered to be more exposed to cardiovascular, gastrointestinal and renal disorders [1]. Therefore, medication that interferes with systemic pathophysiology should be avoided when treating knee OA and local administration of treatments may be recommended. A common example of local administered treatment is intra-articular injections with hyaluronic acid (HA). This option received controversies in recent decades from different authors [13,14,15], but has also been demonstrated to induce superior outcomes with no systemic adverse events compared to oral treatments [16,17]. Endogenous secreted HA is present in the normal synovial fluid and, due to its viscoelastic nature, provides lubricating and shock absorption properties in the joint [18,19].

Besides the highlighted anti-mechanical stress effects due to its molecular structure, other biological mechanisms of action have been described: inflammatory mediators inhibition, chondroprotection and stimulation of endogenous production of proteoglycans and glycosaminoglycans [20,21]. Most recent trials suggested that the effectiveness of intra-articular HA depends on its molecular weight [22,23].

Three main types of HA products are described in the recent literature: HA with an intermediate molecular weight (MW) (500–730 kDa), HA with a high MW (≥1000 kDa) and highly modified HA (cross-linked) [24]. In contrast to others, HA products with a 500–730 kDa MW progress with ease throughout the synovial membrane and co-activate the endogenous synthesis of HA. The resulting largely increased quantity of high-MW HA within the joint theoretically has an additional positive effect [25,26]. 

Recently, new technology was able to provide extended viscoelasticity and residence time to intermediate MW natural HA polymers, while maintaining its bio-tribological features [24]. This technology was enforced in a novel molecule “HYADD^®^4”, introducing a new HA derivative (Hymovis^®^) for sale. Being a new product, its outcomes are not widely published; for that reason, our principal aim was to assess the clinical effectiveness and outcomes of the new HA-based hydrogel Hymovis^®^.

## 2. Materials and Methods

### 2.1. Study Design

A prospective, single-centre, clinical trial was carried out in the Department of Orthopaedics and Traumatology II from the Clinical County Hospital in Tîrgu Mureș, Romania. The accept from the local ethical committee was obtained and all consecutive patients presenting to our outpatient clinic with diagnosed knee OA were screened for inclusion into this study. Radiologically, knee OA had to be Kellgren–Lawrence grade II or III and the VAS pain level on a scale of 0–100 mm had to be above 35 mm. The following criteria of exclusion were considered: pathologies other than primary idiopathic OA, any history of intra-articular injections in the last 6 months, heparin- or platelet-based anti-coagulation treatment in the last month, usage of NSAIDs one week before injection, allergy to HA injections, comorbidities that could affect the outcomes, pregnancy, lactation and infections.

**Figure 1:**
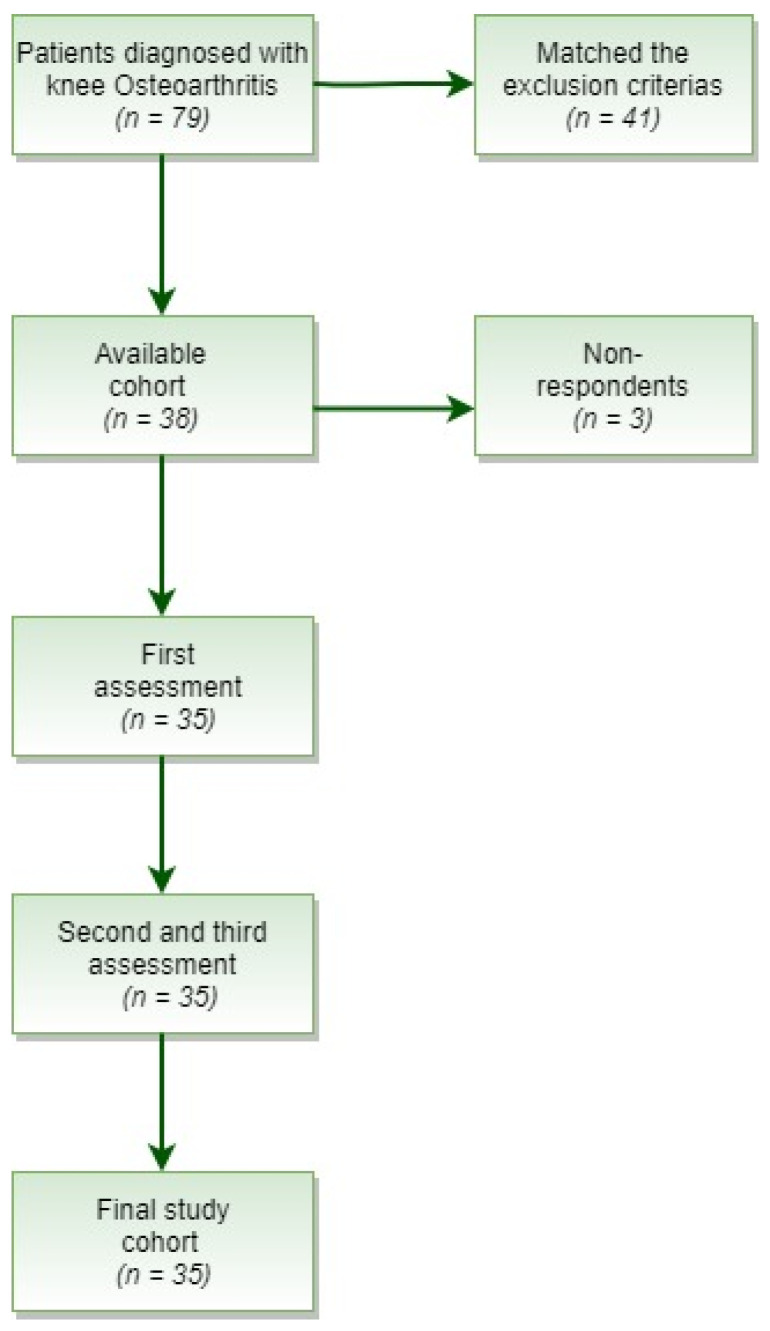
Flow diagram of patient cohort.

A total of 79 patients with primary OA were obtained. Out of those patients, 41 were excluded for matching any of the exclusion criteria (Figure 1). 

**Figure 2:**
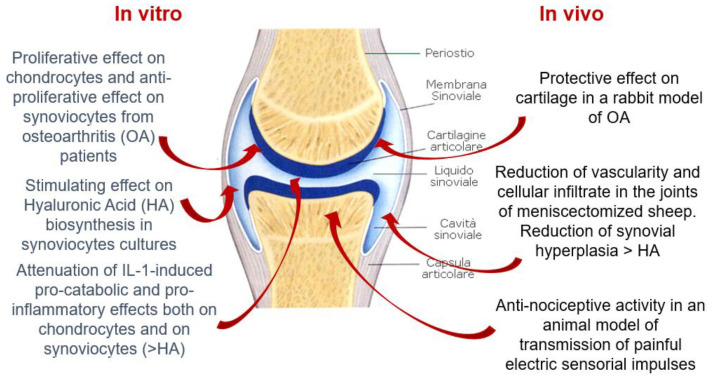
Hymovis. In vitro and in vivo properties.

**Figure 3:**
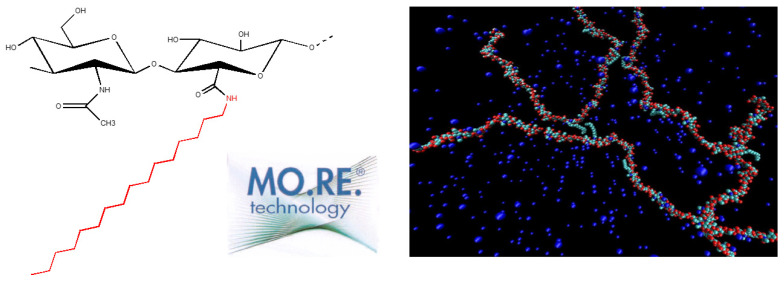
A network stabilized by reversible hydrophilic and hydrophobic interactions, conferring high viscoelasticity and stability to a medium molecular weight (MW) HA derivative.

**Figure 4:**
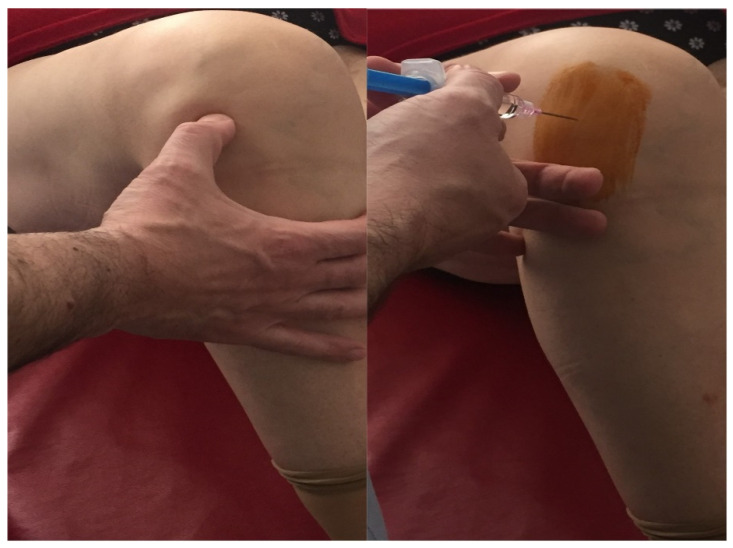
Antero-lateral portal HA injection under routine aseptic conditions.

After signing the informed consent, all patients (*n* = 35) included received two intra-articular doses of 24 mg/3 mL of HA (Hymovis^®^, Fidia Farmaceutici S.p.A, Abano Terme, Italy) with a one-week interval (Figure 2 and Figure 3). The injections were done using the antero-lateral portal under routine aseptic conditions in the outpatient clinic (Figure 4). We used an 18 Gauge 1.20 × 40 mm needle. At baseline time, the subjects were assessed using VAS pain and Western Ontario and McMaster University Osteoarthritis Index (WOMAC) score. Clinical follow-up was performed at 2 and 6 months after the injections.

The Romanian version of the WOMAC 3.1 Index was used for outcome assessment. It is a 24-item survey split into three subscales that measures pain (5 items, score range 0–20), stiffness (2 items, score range 0–8) and physical function (17 items, score range 0–68). The values from each subscale are summed up in order to provide a total normalized score of the index. Every patient completed the WOMAC form before treatment, and at two and six month follow-up visits.

VAS is a substantial and an accepted instrument in calculating the severity of chronic pain [27]. The score consists of a scale that extends from a 0 to 100 mm. The score 0 suggests that the patient feels no pain, while 100 mm on the scale indicates the worst possible pain [28,29]. VAS score was completed along with the WOMAC index at every planned follow-up consultation. Each patient established his pain by sketching a mark on a horizontal line of 100 mm. The distance was then estimated by a study nurse. She was in charge of the patients’ collection of data and evaluation. Three patients were unable to fill the forms necessary for enrollment due to education limitations and were marked as “non-respondents”.

### 2.2. Statistical Analysis

VAS and WOMAC outcomes from each evaluation were compared. The demographic variables comparison was calculated using Chi-square test. The Kolmogorov–Smirnov statistical test for goodness of fit was performed and we obtained a normally distributed population. Data results from WOMAC and VAS were statistically analyzed in GraphPad (InStat) and EpiInfo v 7.1.4.0 (Centers for Disease Control and Prevention, Atlanta, GA, USA) software using *t*-test and repeated ANOVA measurements (single factor). The level of statistical significance was set at *p* < 0.05.

## 3. Results

The final study cohort contained 35 patients. Twenty-five (71%) subjects were females and the mean age at first injection was 63 ± 8 years old. Fourteen patients were smokers for more than one year and 27 had an education level of high school degree or more. Twenty-five individuals reported their employment status as “retired” upon registration. Regarding the body mass index (BMI), two were below 25, one was over 30 and the rest were overweight (25–30). The mean weight was 82.2 kg ± 16.2 SD.

The baseline VAS score was 74.2 ± 11.7 before the treatment. At two-month follow-up, the pain score diminished to 69.6 ± 9.8 with no statistically significant difference from baseline. Mean VAS significantly improved from 69.6 ± 9.8 cm at two months to 57.3 ± 12.1 cm at the end of the study (*p* < 0.0001). 

A slight decrease in the WOMAC pain subscale was recorded after 2 months (*p* = 0.065). At 6 months, a significant improvement regarding the pain parameter was observed compared to baseline (from 10.34 to 7.72; *p* = 0.0004). With regard to the WOMAC stiffness subscale, there were no significant differences from baseline at two months (*p* = 0.819) and six months (*p* = 0.937). The physical function component score improved from baseline at 6 months after injections (Table 1).

There were no important complications through the follow-up. Some minor adverse effects were reported, such as arthralgia and pain at the injected site.

## 4. Discussion

The most important findings of the present study were that Hymovis^®^ infiltrations were proven to have long-term effectiveness in patients with knee OA who had symptomatic pain. As described in other studies, the novel HA-based hydrogel product had positive results in relieving chronic pain in knee OA. A small review of the previously (1998–2012) published papers regarding HA injections is presented in Table 2 [30].

Hymovis is a sterile, apyretic hydrogen produced by HYADD4 (natural sodium hexadecylamide with a high degree of purity, obtained by bacterial fermentation) in buffered isotonic solution. Sodium hyaluronate hexadecylamide confers high viscosity and elasticity by reducing inflammation mediators such as prostaglandin E2, IL1 and IL6. Thus, Hymovis improves the function of lubrication and shock absorption that synovial fluid has, protecting cartilage and soft tissues from mechanical damage. These properties, together with the extended retention time in the joints, allow this product to relieve pain and improve joint function with a short-term treatment regimen [43].

Researchers often perform comparisons between HA and other intraarticular-injected substances. Skwara compared the effectiveness of intra-articular corticosteroids (CS) (triamcinolone) with intraarticular HA by analyzing gait, maximum vertical force, muscle activity and pain [44]. No improvement was reported in either treatment group. However, their data suggest that treatment with hyaluronan can lower the pain and enhance the function of the knee. In 2009, Bannuru conducted a meta-analysis questioning the efficiency of intraarticular HA in the treatment of OA in comparison with CS [45]. The reviews included were published prior to 2004 and only VAS was used as an evaluation indicator. The conclusions of the meta-analysis were in agreement with the results of the present study. Wang et al. stated in a meta-analysis of seven trials that the effectiveness of CS was superior to that of intraarticular HA outcomes at 4 weeks post-injection; in the long term, HA proved its extended efficiency [46]. Leighton et al. compared the two treatments on 441 patients and concluded that efficacy on pain relief is similar in both treatment options and outcomes of the group treated with HA are not inferior to those treated with CS [47]. Conformable to VAS results, HA infiltrations were capable of improving the existent pain in knee OA. The VAS measures on a scale the degree of acute and chronic pain felt by the patients at the moment of the examination. It is a means of evaluating the pain in many published studies that evaluates the treatment efficiency in knee OA [48,49]. HA treatment was proven to offer more prolonged effects in relieving pain compared to the other intra-articular treatments. This is in accordance with other current studies [46,50,51]. The WOMACs Index’s stiffness parameter showed no improvement at any interval after intraarticular HA treatment. Conversely, the pain subscale showed significant improvement two and six months post-intervention with the presented therapy method. Other treatments such as corticosteroids may have a quicker on-set of action regarding pain relief compared to HA but their effect lasted for shorter periods of time. 

It is widely accepted that the placebo effect plays a role in pain relief. There are studies that support this theory. For example, a systematic review published by Gaendam et al., the team suggested that intra-articular saline injections are equally efficient with hyaluronic acid, corticosteroids and platelet rich plasma in the management of hip pain and functional outcomes [52]. Contrary to the findings stated before, there are also multiple published systematic reviews and meta-analyses which suggest that intra-articular hyaluronic acid injection therapy is superior to the placebo injections [53,54,55]. Taken together, we believe that hyaluronic-acid based therapies are more effective and we accept the placebo effect only as an integrated effect.

The WOMAC Index is widely used in assessing outcomes after knee OA therapy. The WOMAC Index has its validity and responsiveness correctly scaled when used together with VAS, making for accurate results when combined [56]. The notable importance of the therapy is the extent of the pain relief effect. As mentioned previously, HA has a longer effectiveness in reducing pain compared to other IA-injected substances. The considerably higher cost of HA constitutes a limitation to the extensive implementation of this therapy. There are no concise studies evaluating cost-efficiency for the presented therapy. In accordance with our results, we suggest that HA can be administered every six months in order to provide extended pain relief. Furthermore, multiple administrations may lengthen the period until prosthetic knee replacement is needed. There are some limitations to our study design. There was no comparison group, and only short-term outcomes were reported. Additionally, we consider our patient sample size a limitation, compared to other trials to date. As the injection costs were partially funded by a private pharmaceutical company, financial restrictions were considered limitations for the presented paper. Our results confirmed the efficacy of the novel HA-based hydrogel (Hymovis^®^) regarding pain relief in patients suffering from knee OA. Clinical results proved its ability to reduce pain and improve joint function.

## Figures and Tables

**Table 1:** Western Ontario and McMaster University Osteoarthritis Index (WOMAC) subscale scores by follow-up time.

	WOMAC Score per Subscale		*p* Value Comparison from Baseline
	Mean	SD	
Pain before (baseline)	10	3	-
Pain after 2 months	9	2	n.s. *
Pain after 6 months	8	1	0.0004
Stiffness before (baseline)	3	1	-
Stiffness after 2 months	3	1	n.s.
Stiffness after 6 months	3	1	n.s.
Physical function before (baseline)	30	8	-
Physical function after 2 months	29	7	n.s.
Physical function after 6 months	25	5	0.0025

* not significant.

**Table 2:** Published papers (1998–2012) regarding HA injections in knee OA treatment with outcomes measured by WOMAC form.

Author & Year	Study Population	Treatment	Outcomes
Altman et al., 1998 [31]	495	Group 1 (*n* = 115): placebo Group 2 (*n* = 105): Hyalgan 20 mg/2 mL (500–700 kDa) Group 3 (*n* = 113): NSAID	WOMAC physical function: Hyalgan improvement compared to placebo—*p* = 0.047
Brandt et al., 2001 [32]	226	Group 1 (*n* = 112): placebo Group 2 (*n* = 114): Orthovisc 2 mL, 15 mg/mL (1000–2900 kDa—high molecular weight)	WOMAC function Group 1: mean change from baseline: −9.8 Group 2: mean change from baseline: −14.7 *p* = 0.02
Karlsson et al., 2002 [33]	246	Group 1 (*n* = 66): placebo Group 2 (*n* = 92): Artzal (2.5 mL 1% hyaluronan; 1000 kDa) Group 3 (*n* = 88): Synvisc (2 mL 0.8%; 7000 kDa)	WOMAC physical function Group 1: mean change from baseline: −11.1 Group 2: mean change from baseline: −7.3 Group 3: mean change from baseline: −11.7 Group 3 vs. 2: standard mean difference −0.297
Petrella et al., 2002 [34]	120	Group 1 (*n* = 28): placebo Group 2 (*n* = 25): Suplasyn (molecular weight not reported) Group 3 (*n* = 29): Suplasyn + NSAID Group 4 (*n* = 26): NSAID	WOMAC Disability Group 1: mean change from baseline: −0.99 Group 2: mean change from baseline: −1.65 Group 3: mean change from baseline: −1.17 Group 4: mean change from baseline: −1.56 Standard mean difference: −0.234
Kahan et al., 2003 [35]	506	Group 1 (*n* = 253): conventional treatment Group 2 (*n* = 253): Synvisc (molecular weight not reported)	WOMAC function Group 1: mean change from baseline: −7 Group 2: mean change from baseline: −18.4 Standard mean difference: −0.567
Blanco et al., 2008 [36]	42	Group 1 (*n* = 20): placebo Group 2 (*n* = 22): Adant (900 kDa)	WOMAC physical function Group 1: mean change from baseline: −4.4 Group 2: mean change from baseline: −24.7 Standard mean difference: −1.080
Raman et al., 2008 [37]	392	Group 1 (*n* = 199): Synvisc (Hylan GF 20 – 6000 kDa) Group 2 (*n* = 193): Hyalgan (500–730 kDa)	WOMAC physical activity Group 1: mean change from baseline: −21.8 Group 2: mean change from baseline: −6.8 Standard mean difference: −0.882
Huang et al., 2011 [38]	200	Group 1 (*n* = 100): Placebo Group 2 (*n* = 100): Hyalgan (20 mg/2 mL)	WOMAC function Group 1: mean change from baseline: −18.2 Group 2: mean change from baseline −25.16 Standard mean difference: −0.415
Pavelka et al., 2011 [39]	381	Group 1 (*n* = 192): Synovial (800−1200 kDa) Group 2 (*n* = 188): Synvisc (6000 kDa)	WOMAC function Group 1: mean change from baseline 3.9 Group 2: mean change from baseline 3.4 Standard mean difference: −0.009
Berenbaum et al., 2012 [40]	426	Group 1 (*n* = 209): Hyalgan (500−730 kDa) Group 2 (*n* = 217): GO-ON (2.5 mL, 10 mg/mL; 800–1500 kDa)	WOMAC function Group 1: mean change from baseline: −15.4 Group 2: mean change from baseline: −22.2 Standard mean difference: −0.326
DeCaria et al., 2012 [41]	30	Group 1 (*n* = 15): Placebo/acetaminophen Group 2 (*n* = 15): Hyaluronic acid (2 mL, 20mg/mL; 730 kDa)	WOMAC function Group 1: mean change from baseline: −3.53 Group 2: mean change from baseline: −9.07 Standard mean difference: −0.586
Khanasuk et al., 2012 [42]	32	Group 1 (*n* = 15): Synvisc (Hylan GF-20; single 6ml injection; high molecular weight) Group 2 (*n* = 15): Hyalgan (single injection; low molecular weight)	WOMAC function Group 1: mean change from baseline: −20 Group 2: mean change from baseline: −22 Standard mean difference: 0.053
